# Effect of *Citrus bergamia* Supplementation on Body Composition in Humans: A Systematic Review and Meta‐Analysis of Randomized Controlled Trials

**DOI:** 10.1111/obr.70094

**Published:** 2026-01-22

**Authors:** Carmelo Pujia, Yvelise Ferro, Alberto Castagna, Elisa Mazza, Samantha Maurotti, Francesca Rita Noto, Valeria Rizzo, Tiziana Montalcini, Arturo Pujia

**Affiliations:** ^1^ O.U. Clinical Nutrition Renato Dulbecco Hospital Catanzaro Italy; ^2^ Department of Medical and Surgical Sciences University “Magna Græcia” of Catanzaro Catanzaro Italy; ^3^ Department of Clinical and Experimental Medicine University “Magna Græcia” of Catanzaro Catanzaro Italy; ^4^ Research Center for the Prevention and Treatment of Metabolic Diseases University “Magna Græcia” Catanzaro Italy

**Keywords:** bergamot, body composition, *Citrus bergamia*, meta‐analysis, nutraceuticals, obesity, polyphenols, weight management

## Abstract

This systematic review and meta‐analysis of randomized controlled trials (RCTs) was conducted to assess the effect of nutraceuticals containing *Citrus bergamia* on the management of obesity in adults by assessing measures related to obesity. We searched the following databases until October 2024: PubMed, Web of Science, Scopus, and the Cochrane Central Register of Controlled Trials. The inclusion criteria were RCTs involving adults (≥ 18 years), the use of nutraceuticals containing *C. bergamia* , and an intervention duration of ≥ 4 weeks. Data extraction focused on weight loss, body mass index, waist circumference, waist‐to‐hip ratio, and body fat percentage, and the risk of bias was assessed using the Revised Cochrane Risk‐of‐Bias Tool. Out of 966 reports, 11 RCTs were eligible for inclusion in the meta‐analysis. The pooled results, using a random‐effects model, showed that *C. bergamia* supplementation significantly decreased body weight (SMD: −0.64; 95% CI: −1.15, −0.13; *p* = 0.01; *I* ^2^ = 90%), body mass index (SMD: −0.85; 95% CI: −1.35, −0.35; *p* = 0.0008; *I* ^2^ = 90%), and waist circumference (SMD: −0.41; 95% CI: −0.65, −0.16; *p* = 0.001; *I* ^2^ = 49%). However, no significant effect of bergamot‐containing nutraceuticals was observed on the other body composition parameters analyzed. Subgroup analysis suggested that bergamot intake significantly changed anthropometric parameters for periods of ≤ 12 weeks, at dosages of ≤ 600 mg/day, and among participants with overweight and obesity. Overall, the current meta‐analysis suggests that nutraceuticals containing *C. bergamia* show promise in the prevention and management of obesity.

**Trial Registration:**

International Prospective Register of Systematic Reviews (PROSPERO): CRD42023465541 (https://www.crd.york.ac.uk/prospero/display_record.php?RecordID=465541)

## 1 Introduction

Obesity is a multifactorial disease defined as abnormal or excessive fat accumulation that presents a risk to health because it is a major risk factor for several noncommunicable diseases (NCDs), such as cardiovascular diseases, diabetes, and several types of cancer [1]. It is a major public health concern that has reached pandemic proportions worldwide. In 2022, over one million people globally were affected by obesity [2], with current estimates indicating that this number could rise to four billion by 2035, representing over 51% of the world's adult population [3].

In Italy, the situation is equally alarming, with 33% of adults classified as having overweight and more than 10% as having obesity [4]. Obesity rates are also rising, with approximately 30% of children being classified as having overweight or obesity [5]. Furthermore, obesity and its associated health problems have a significant economic impact on the global healthcare system [3, 6].

Therefore, obesity represents a growing public health challenge worldwide, and effective strategies for both prevention and management of obesity are urgently needed. It is well known that a weight loss of between 5% and 10% is sufficient to achieve clinically significant improvements in health risk factors [7]. To achieve successful maintenance of weight loss over time, changes in lifestyle including physical exercise and a diet that reduces excessive energy intake and improves dietary quality were recommended [8, 9]. Although substantial weight loss is possible through a wide range of treatment modalities, long‐term maintenance of lost weight is much more challenging and weight regain is typical. In a meta‐analysis of 29 long‐term weight loss studies, more than half of the weight lost was regained within 2 years, and more than 80% of the weight lost was regained within 5 years [10]. Furthermore, 80% of people with obesity fail to lose weight long term with diet and exercise alone [10]. While lifestyle modifications such as diet and exercise are foundational, there is growing interest in the potential therapeutic role of natural products, particularly polyphenol‐rich plant extracts, in managing obesity and its metabolic consequences [11, 12].

Bergamot ( *Citrus bergamia* Risso et Poiteau), a citrus fruit native to the Calabria region in southern Italy, has garnered considerable attention for its potential health benefits. Rich in polyphenols, flavonoids, and other bioactive compounds, bergamot has demonstrated promising effects in improving lipid profiles and reducing oxidative stress, making it a potential adjunct therapy in managing metabolic disorders such as obesity and dyslipidemia [13, 14].

Several studies have highlighted the lipid‐lowering, anti‐inflammatory, and antioxidant properties of bergamot extracts, particularly bergamot polyphenolic fraction (BPF), in improving metabolic health markers [14, 15, 16].

Preclinical and clinical studies suggest that supplementation with *C. bergamia* extract can reduce body weight and total fat mass [17, 18]. However, despite this evidence, to date, no clinical studies have been conducted to evaluate the effects of *C. bergamia* on body weight loss. This review aims to examine the existing evidence on the effects of bergamot on weight management. Specifically, we conducted a meta‐analysis of randomized controlled trials (RCTs) to evaluate the impact of nutraceuticals containing *C. bergamia* on body weight and body composition changes in adults. The findings from this analysis may provide new insights into the role of natural compounds such as bergamot in the prevention and treatment of obesity and its related complications.

### 1.1 Selection and Search Strategy

This review was conducted following the guidelines of the Preferred Reporting Items for Systematic Review and Meta‐Analysis (PRISMA) [19]. The protocol for the study was registered with the International Prospective Register of Systematic Reviews (PROSPERO; CRD42023465541 accessed on October 2, 2023) (https://www.crd.york.ac.uk/prospero/display_record.php?RecordID=465541). The guidelines in the Cochrane Handbook for Systematic Reviews of Interventions were used to conduct this meta‐analysis [20].

We conducted a search for relevant articles published through four databases, including PubMed, Web of Science, Scopus, and Cochrane Central Register of Controlled Trials (CENTRAL), without any time or language restrictions. Our search was focused on those studies in humans that explore the effect of *C. bergamia* on body composition. The review question was: “Has *C. bergamia* supplementation a significant effect on body composition in humans?”

The search strategy included free‐text terms and subject headings related to intervention ( *C. bergamia* and Bergamot). Search strategies for PubMed and Embase were combined with dedicated RCT filters proposed by the Cochrane Collaboration.

The studies were searched using specific terms related to bergamot and obesity (Bergamot OR *Citrus bergamia* OR Bergamot orange) AND (intervention OR intervention* OR trial OR randomized OR random OR randomly OR placebo OR assignment OR “clinical trial” OR RCT OR “Clinical Trials as Topic” OR cross‐over OR parallel).

The last search was conducted from the inception of the electronic databases up until October 21, 2024.

### 1.2 Inclusion/Exclusion Criteria

This review was designed to evaluate the effectiveness of *C. bergamia* supplementation on weight loss and changes of body composition. It included only RCTs with either a parallel or crossover design, where the intervention lasted at least 1 month and was conducted on adult individuals (mean age ≥ 18 years), regardless of health status. This also included adults at risk for chronic diseases (e.g., obesity, mild hypercholesterolemia, metabolic syndrome, prediabetes, or hypertension).

RCTs were eligible if they examined Bergamot supplementation either as a single active compound or as part of a nutraceutical formulation containing other bioactive molecules. Only studies comparing the nutraceutical with a placebo, with another nutraceutical, or with a drug were also included. Therefore, all studies were included in which the nutraceutical containing *C. bergamia* was administered in the form of capsule, pill, powder, solution, and tablet. Finally, we also included studies that reported changes in body weight and/or changes in other outcomes (i.e., body mass index [BMI], waist circumference [WC], waist‐to‐hip ratio [WHR], and fat mass [FM]) between the intervention and control groups during the treatment period or that sufficient information could be obtained to estimate such values.

For this review, the exclusion criteria were as follow: (1) in vitro or animal studies; (2) studies lacking an active control group; (3) studies where bergamot was not administered as a nutraceutical; and (4) RCTs conducted on children, adolescents, pregnant, or breastfeeding women. In addition, trials that included dietary or exercise cointerventions not equally applied to both groups were also excluded. Systematic reviews and meta‐analyses performed to examine the effect of bergamot supplementation on lipid profiles were examined to identify additional studies not captured in the initial database search. All extracted articles were reviewed, and the titles and abstracts were screened by two independent researchers (E.M. and S.M.) using the Rayyan application for systematic reviews to remove duplicates and assess eligibility based on the inclusion criteria. Disagreements between the two investigators were adjudicated by the third investigator or by group consensus. Each rejected article was reviewed by a third investigator (A.P.) to confirm or refute its exclusion. Finally, the full texts of all articles were read independently by the same authors and further discussed.

### 1.3 Data Extraction and Quality Assessment

Data extraction was independently carried out by two researchers (C.P. and Y.F.) using an Excel spreadsheet. Once the definitive articles have been included, we created a standardized form to extract the following data: (1) first author's surname; (2) year of publication; (3) country of study; (4) study design (parallel or crossover); (5) number and type of participants enrolled; (6) mean age of participants; (7) characteristics of the intervention and control groups; (8) intervention duration, dosage, and type of nutraceutical and comparator; and (9) study outcomes (changes of body composition postintervention [weight, BMI, WC, WHR, and FM]) reported as mean (standard deviation [SD]), mean (standard error [SE]), or mean difference with 95% confidence intervals (CIs). The quality of the RCTs included in the meta‐analysis was independently assessed using the Revised Cochrane Risk‐of‐Bias Tool for Randomized Trials (RoB 2). Based on the study methodology, the quality of the studies was rated as low, unclear, or high risk of bias. The following domains were assessed to determine study quality: randomization process, allocation concealment, blinding of participants and personnel to the interventions, blinding of outcome assessment, handling of missing data, reporting of endpoint outcomes, and other bias [21].

### 1.4 Statistical Analysis

The meta‐analysis was conducted using all studies that were homogeneous in terms of interventions, reported outcomes, and that included information on the endpoints analyzed in this study. The primary outcome of this meta‐analysis was the change in body weight (kg). Secondary outcomes included changes in BMI, WC, and, where applicable, changes in WHR and FM. Data reported as SE and CI were converted to SD [20]. Additionally, if body weight was reported in pounds, it was converted to kilograms. The effect size on weight and for secondary outcomes was estimated using the standardized mean difference (SMD). All analyses were performed using random‐effects models. We performed subgroup analyses by considering study duration (≤ 12 weeks), dose (≤ 600 mg/day vs. ≥ 1000 mg/day), and participants with overweight or obesity (mean BMI ≥ 25 kg/m^2^). Heterogeneity between studies was evaluated using the *χ* ^2^ test and quantified using the *I* ^2^ inconsistency measure, where values of 25%, 50%, and 75% correspond to low, moderate, and high heterogeneity, respectively [22]. Substantial heterogeneity was considered for an *I* ^2^ value greater than 50% [23]. Furthermore, funnel plots were used to assess study precision and systematic heterogeneity. Review Manager Software (Version 5.4; Cochrane Training) was used to perform all statistical analyses.

## 2 Results

**FIGURE 1:**
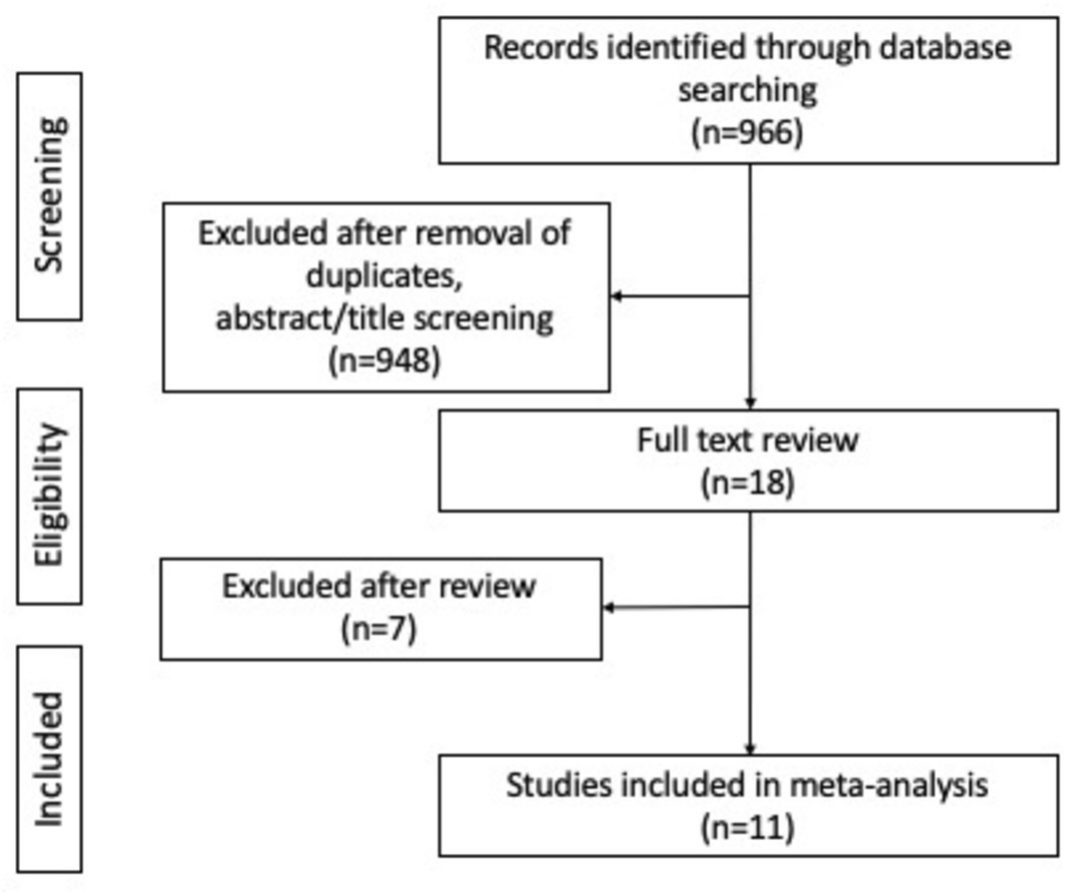
PRISMA flow‐chart research strategy.

Considering the four databases, we found 966 articles, 948 were excluded after removing duplicates and screening their title and abstract (Figure 1). The full text of 18 articles was assessed for eligibility. Of those, one article was excluded because the intervention duration was only 2 weeks [24]. Another study was excluded because, despite randomizing 107 patients into two groups (placebo and bergamot nutraceutical), the authors did not present control group results or compare any parameters between the placebo and intervention groups [25]. Two studies were excluded as two did not have the outcomes of interest (weight, BMI, WC, WHR, and FM) [26, 27], while one lacked available results related to the outcomes of interest [28]. Finally, two studies were excluded because the control groups followed only a dietary intervention and did not receive any type of placebo [29, 30, 31, 32, 33, 34, 35, 36, 37, 38, 39]. A total of 11 studies were included in this meta‐analysis [30, 31, 32, 33, 34, 35, 36, 37, 38, 40] (Figure 1).

The main characteristics of the included studies, the types of interventions are reported in Table 1. A total of 11 studies published between 2011 and 2024 were included. Most studies represented both genders, with a higher enrollment of females. The studies included a total of 1088 adults. Sample sizes varied from 25 to 237 participants. The mean age of adults enrolled in the 11 studies ranged from 44 to 65 years, with an average BMI between 22.8 and 30.8 kg/m^2^. Of the 11 studies, two were conducted in participants with nonalcoholic fatty liver disease [36, 37], four assessed participants with mixed hyperlipidemia [30, 33, 35, 38], and four participants with hypercholesterolemia [30, 31, 32, 34, 40]. One study was conducted in adults with metabolic syndrome [40]. The dropout rates ranged from 2% to 16%, with no dropouts reported in seven studies [30, 31, 32, 34, 35, 38, 41]. For the active treatments, three studies evaluated the exclusive effect of *C. bergamia* extracts, administered at a daily dose ranging from 400 to 1500 mg [31, 34, 38]. Six studies evaluated the effects of a formulation that included bergamot extracts combined with artichoke extracts [30, 32, 35, 36, 37, 41], in particular four of these also included vitamins or other bioactive molecules in the formulation [30, 35, 37, 41]. Finally, two studies involved the use of *C. bergamia* extracts combined with other plant extracts [33, 40]. In 10 studies, the control group was given a placebo, while in one study, the control group took statins [38]. The duration of intervention ranged from 4 to 24 weeks. In seven studies, no significant differences in weight loss were reported [30, 31, 32, 34, 35, 37, 41], while other studies observed significant reductions in body weight in the intervention group compared with the control group [33, 36, 40].

**TABLE 1:** Characteristics of studies included in the meta‐analysis.

Study	Study design and duration (weeks)	Type of participants (number of subjects)	BMI	Intervention dose (mg)	Outcomes	Results on body composition
			Mean ± SD (kg/m^2^)			
Mollace et al., 2011 (Italy) [38]	Multiple‐arm, double‐blind, randomized, placebo‐controlled study (4 weeks)	Adults with mixed hyperlipidemia with or without hyperglycemia (n. 237)	Not reported	500–1500 mg of BPF/daily	Lipid and glucose parameters	Differences in body composition after treatment between groups not reported
Cai et al., 2017 (China) [33]	Randomized, double‐blind, placebo‐controlled trial (12 weeks)	Older adults with mixed hyperlipidemia (n. 98)	25.97 ± 3.25	500 mg of *Citrus bergamia* extracts plus plant sterol esters and vitamins 4 capsules/daily	Serum cholesterol and body weight	Significant reduction in weight, BMI and WC in the group that took bergamot compared with placebo
Cicero et al., 2019 (Italy) [35]	Three‐arm, randomized, double‐blind, placebo‐controlled clinical trial (24 weeks)	Subjects with dyslipidemia and overweight (n. 90)	26.77 ± 1.8	120 mg flavonoids of Bergamot extract phytosterols, artichoke extract and vitamin C 1 capsule/daily	Lipid and glucose parameters	No significant differences in body composition between groups were observed after treatment
Ferro et al., 2020 (Italy) [36]	Randomized, double‐blind, placebo‐controlled trial (12 weeks)	Adults with NAFLD (n. 102)	29.0 ± 3.5	300 of BPF plus artichoke extract 1 capsule/daily	Liver fat content	Significant reduction in weight and BMI in the group that took BPF compared with placebo
Hancke et al., 2021 (India) [40]	Randomized, double‐blind, placebo‐controlled trial (16 weeks)	Adults with overweight or obesity and mild hypercholesterolemia (n. 97)	30.86 ± 2.42	200–400 mg of *C. bergamia* and *Eurycoma longifolia* 3 capsules/daily	Body composition and lipid parameters	Significant reduction in BMI in the treatment group compared with placebo
Rondanelli et al., 2021 (Italy) [31]	Randomized, double‐blind, placebo‐controlled trial (12 weeks)	Adults with overweight or obesity and mild hypercholesterolemia (n. 64)	28.22 ± 3.18	500 mg of bergamot phytosome 2 capsules/daily	Visceral adipose tissue and metabolic profile	Significant reduction in VAT, weight and fat mass within treatment group pre–post supplementation
Riva et al., 2021 (Italy) [32]	Randomized, double‐blind, clinical trial (8 weeks)	Adults with overweight and mild hypercholesterolemia (n. 60)	27.85 ± 2.87	600 mg of bergamot phytosome plus artichoke extract	Metabolic profile and body composition	Significant reduction in WC and VAT between groups and in weight, BMI and fat mass within treatment group
Ferro et al., 2022 (Italy) [37]	Randomized, double‐blind, placebo‐controlled clinical trial (12 weeks)	Adults with NAFLD (n. 140)	28.9 ± 4	300 mg of BPF plus artichoke extract and other bioactive molecules 6 capsules/daily	Liver fat content	Significant reduction in BMI and weight within treatment group pre–post supplementation
Fogacci et al., 2022 (Italy) [30]	Randomized, double‐blind, placebo‐controlled study (8 weeks)	Adults with polygenic hypercholesterolemia (n. 60)	24.1 ± 3.4	1000 mg of phytosome BPF plus artichoke extract and vitamins 1 capsule/daily	Lipid parameters, systemic inflammation, and indexes of NAFLD	Significant reduction in WC in the group that took BPF compared with placebo
Pierdomenico et al., 2023 (Italy) [34]	Parallel arms, randomized, double‐blind, placebo‐controlled clinical study (12 weeks)	Adults with moderate hypercholesterolemia (n. 50)	22.8 ± 2.2	400 mg of bergamot extract 1 capsule/daily	Lipid and glucose parameters	No significant differences in body composition between groups were observed after treatment
Fogacci et al., 2024 (Italy) [41]	Randomized, double‐blind, placebo‐controlled clinical trial (12 weeks)	Adults with dyslipidemia (n. 90)	26.85 ± 1.8	375 bergamot dry extract, plus artichoke extract and other bioactive molecules 1 capsule/daily	Lipid and glucose parameters and indexes of NAFLD	No significant differences in BMI between groups were observed after treatment

Abbreviations: BMI, body mass index; BPF, bergamot‐derived polyphenolic fraction; NAFLD, nonalcoholic fatty liver disease; VAT, visceral adipose tissue; WC, waist circumference.

**FIGURE 2:**
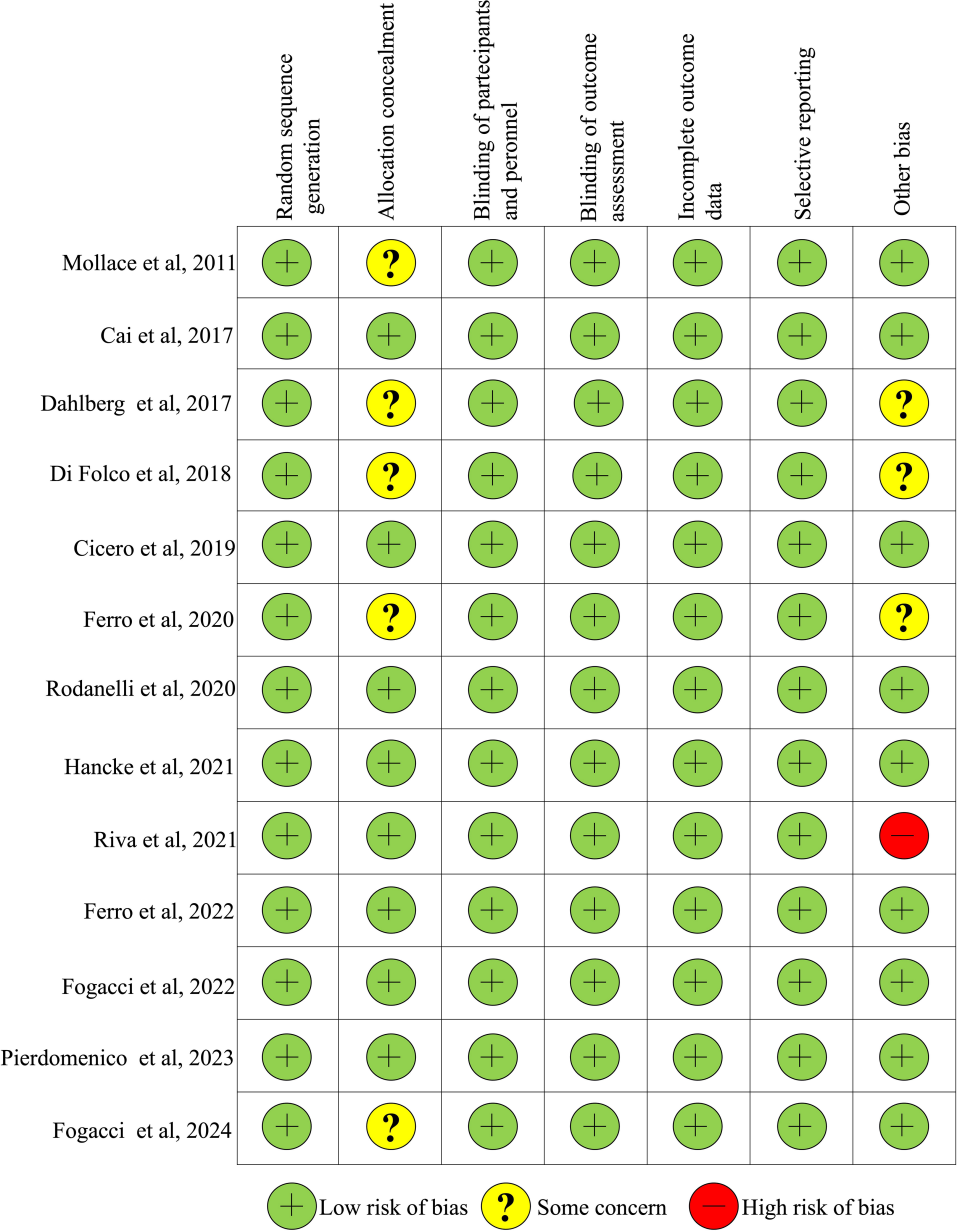
Assessment of the methodological quality of included studies.

Figure 2 shows the risk of bias of included studies. Of the 11 studies, eight were classified as having a low risk of bias across all evaluated domains. Two studies demonstrated an uncertain risk of bias related to selection bias [36, 38], specifically concerning allocation concealment. Only, one study was classified as high risk of bias [32].

**FIGURE 3:**
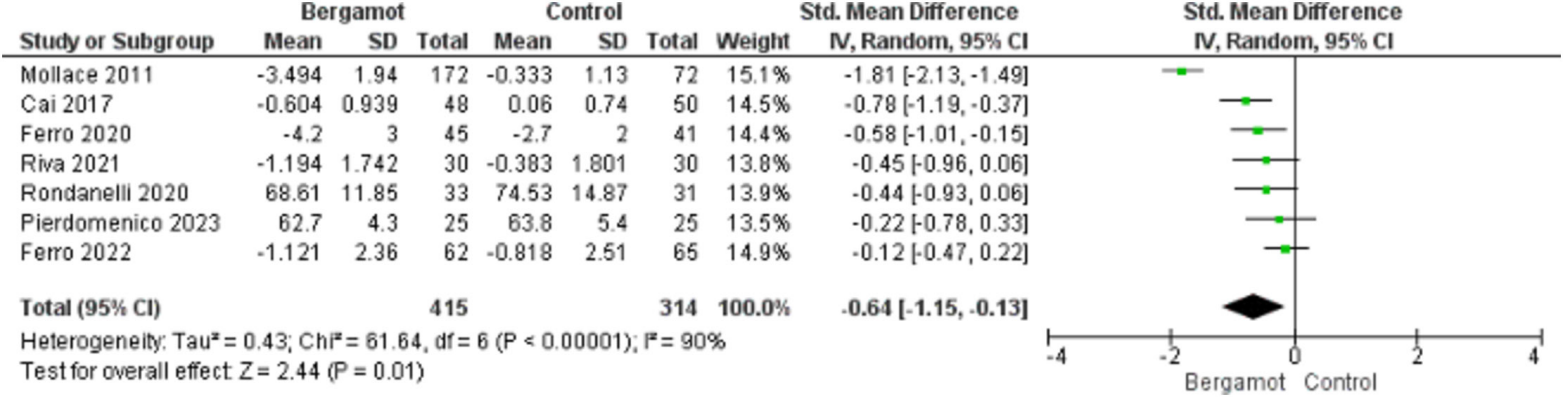
Forest plot of the effects of bergamot‐containing nutraceuticals on body weight, with intervention durations ranging from 4 to 12 weeks.

Figure 3 shows the meta‐analysis results for body weight at 12 weeks. For body weight, seven studies presented data for quantitative analysis. Total participants included ranged from 25 to 172 in intervention group and from 25 to 65 in control group. Significant effect of *C. bergamia* supplementation was observed for body weight (SMD: −0.64; 95% CI: −1.15, −0.13; *p* = 0.01; *I* ^2^ = 90%) (Figure 3).

**FIGURE 4:**
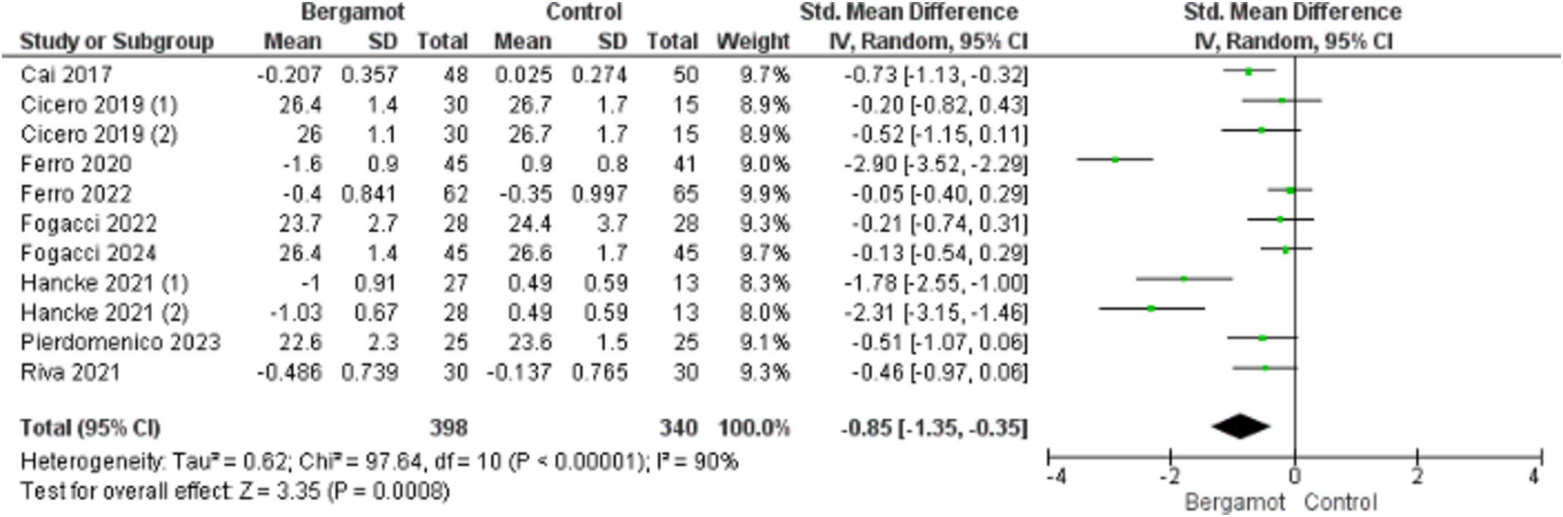
Forest plot of the effects of bergamot‐containing nutraceuticals on BMI, with intervention durations ranging from 4 to 24 weeks.

Figure 4 shows the results of meta‐analysis for BMI at 24 weeks. Nine studies reported data for quantitative analysis. The number of participants included ranged from 25 to 62 in intervention group and from 25 to 65 in control group. Significant effect of bergamot supplementation was observed for BMI (SMD: −0.85; 95% CI: −1.35, −0.35; *p* = 0.0008; *I* ^2^ = 90%) (Figure 4).

**FIGURE 5:**
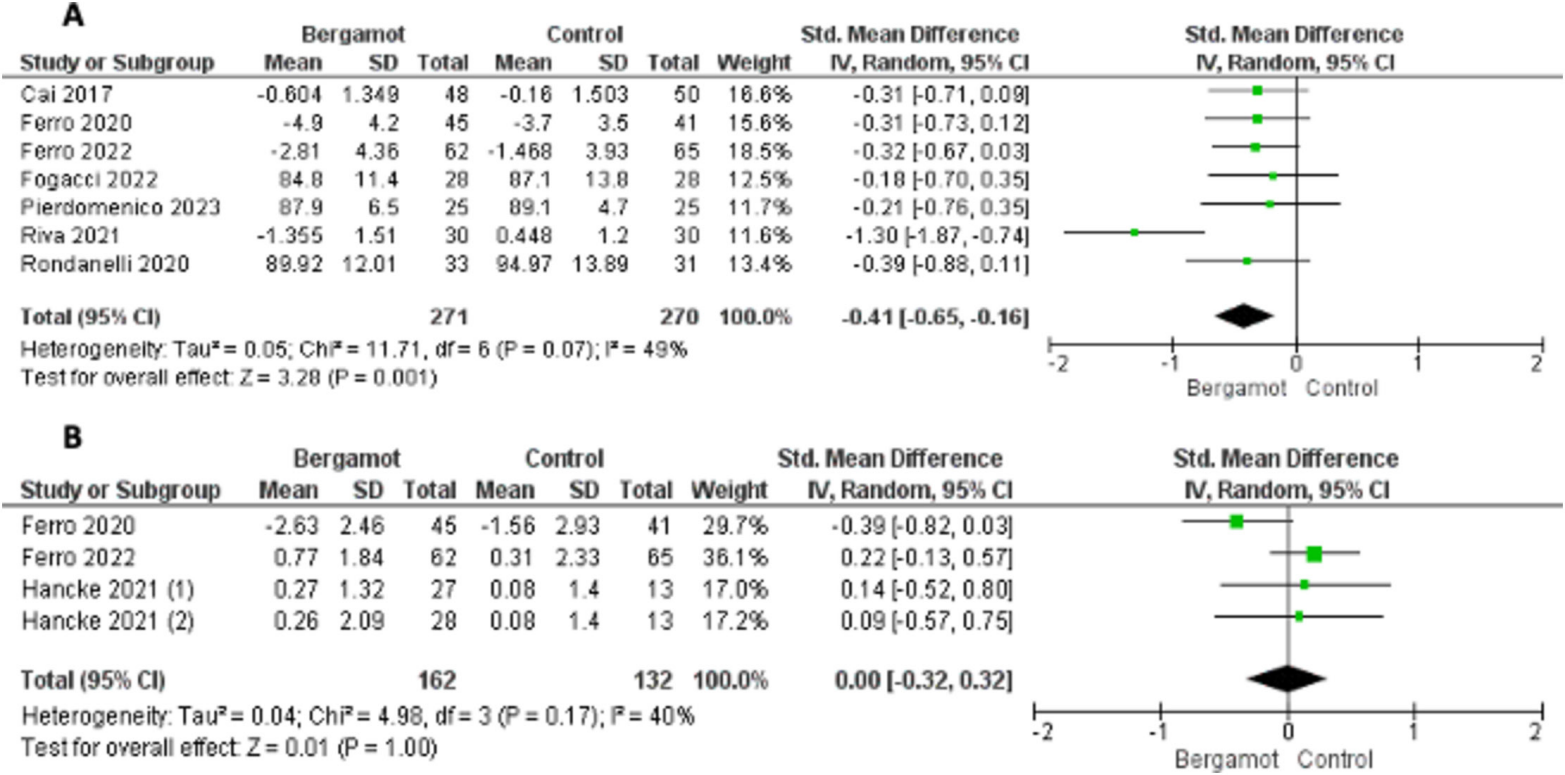
Forest plot of the effects of bergamot‐containing nutraceuticals on waist circumference, with intervention durations ranging from 8 to 12 weeks, and on body fat mass, with intervention durations ranging from 12 to 16 weeks.

Figure 5 shows the meta‐analysis results for WC and FM. For WC, there was a significant effect after treatment (SMD: −0.41; 95% CI: −0.65, −0.16; *p* = 0.001; *I* ^2^ = 49%) (Figure 5A). No significant effect of bergamot nutraceuticals was observed for FM percentage (SMD: 0.00; 95% CI: −0.32, 0.32; *p* = 1.00; *I* ^2^ = 40%) (Figure 5B).

Subgroup analysis for intervention duration and *C. bergamia* dosage on body weight, BMI, WC, WHR, and FM are presented in Table 2. Table 2 also presents the subgroup analysis for participants with overweight and obesity (mean BMI ≥ 25 kg/m^2^). Significant effects of the bergamot‐containing nutraceuticals intake on body weight, BMI, and WC were observed in studies with an intervention duration of ≤ 12 weeks. However, no significant effects were found for changes in WHR and FM (Table 2). Moreover, significant effects of the bergamot‐containing nutraceuticals intake at dosages of ≤ 600 mg/day on body weight, BMI, and WC were observed in studies. However, no significant effects were found for changes in WHR and FM (Table 2). Finally, *C. bergamia* supplementation significantly reduces body weight, BMI, and WC in participants with overweight and obesity but not WHR and FM percentage (Table 2).

**TABLE 2:** Subgroup analysis of the effects of bergamot‐containing nutraceuticals on each outcome categorized by intervention duration, dosage, and body mass index.

	Participants (n)	SMD [95% CI]	*p*	*I* ^2^ (%)
Length of follow‐up 4–12 weeks				
Body weight (kg)	729	−0.64 [−1.15, −0.13]	0.01	90
BMI (kg/m^2^)	657	−0.60 [−1.09, −0.10]	0.02	89
Waist circumference (cm)	541	−0.41 [−0.65, −0.16]	0.001	49
WHR	311	0.00 [−0.25, 0.25]	0.99	20
Fat mass (%)	213	−0.08 [−0.67, 0.52]	0.81	79
Dosage ≤ 600 mg/day Length of follow‐up 4–24 weeks				
Body weight (kg)	435	−0.71 [−1.13, −0.30]	0.0008	79
BMI (kg/m^2^)	555	−1.03 [−1.62, −0.43]	0.0008	90
Waist circumference (cm)	294	−0.51 [−0.96, −0.06]	0.03	72
WHR	184	−0.13 [−0.42, 0.16]	0.37	0
Fat mass (%)	227	−0.23 [−0.51, 0.05]	0.11	8
Dosage ≥ 1000 mg/day Length of follow‐up 4–24 weeks				
Body weight (kg)	366	−1.09 [−2.73, 0.56]	0.19	98
BMI (kg/m^2^)	183	−0.10 [−0.39, 0.19]	0.49	0
Waist circumference (cm)	247	−0.31 [−0.56, −0.05]	0.02	0
WHR	/	/	/	/
Fat mass (%)	/	/	/	/
BMI ≥ 25 kg/m^2^ Length of follow‐up 4–24 weeks				
Body weight (kg)	435	−0.46 [−0.70, −0.22]	0.0002	35
BMI (kg/m^2^)	632	−0.97 [−1.57, −0.37]	0.002	92
Waist circumference (cm)	435	−0.49[−0.80, −0.17]	0.003	62
WHR	311	0.00 [−0.25, 0.25]	0.99	20
Fat mass (%)	294	0.00 [−0.32, 0.32]	1	40

Abbreviations: BMI, body mass index; CI, confidence interval; SMD, standardized mean difference; WHR, waist‐to‐hip ratio.

Figure S1 reported the visual inspection of funnel plots to assess any potential publication bias of the studies included.

## 3 Discussion

This review is the first to evaluate the data in the literature on the efficacy of nutraceutical supplementation containing *C. bergamia* for the management of obesity. It provides a comprehensive analysis of anthropometric parameters related to obesity in adult subjects. The main result of this meta‐analysis clearly showed that supplementation with *C. bergamia* is significantly effective in reducing body weight (SMD: −0.64; 95% CI: −1.15, −0.13; *p* = 0.01; *I* ^2^ = 90%) (Figure 3), BMI (SMD: −0.85; 95% CI: −1.35, −0.35; *p* = 0.0008; *I* ^2^ = 90%) (Figure 4) and WC (SMD: −0.41; 95% CI: −0.65, −0.16; *p* = 0.001; *I* ^2^ = 49%) (Figure 5A) in treatments lasting between 4 and 24 weeks. However, the study found no significant effect on FM percentage (SMD: 0.00; 95% CI: −0.32, 0.32; *p* = 1.00; *I* ^2^ = 40%) (Figure 5B).

These results suggest that dietary supplementation with *C. bergamia* may have an effect in the prevention of obesity. Based on the estimated baseline body weight and SD, the pooled effect size for weight reduction (SMD = −0.64) corresponds to an average weight loss of approximately 7.1 kg or 9.8% of basal body weight. A weight loss in the range of 5%–10% is well known to produce clinically significant improvements in cardiometabolic risk factors [7]. Therefore, the observed effects of nutraceutical supplementation containing *C. bergamia* may be not only statistically significant but also clinically relevant.

However, as shown in Figure S1, the funnel plots were not symmetrical, particularly for body weight reduction (Figure S1A) and BMI reduction (Figure S1B), suggesting the potential presence of publication bias or small‐study effects. In contrast, the funnel plot for WC (Figure S1C) appeared more symmetrical, indicating a lower likelihood of systematic bias. The funnel plot for FM percentage (Figure S1D) could not be reliably interpreted due to the limited number of studies included. Therefore, the effect size of the interventions should be interpreted with caution.

On the other hand, the subgroup analysis confirmed the efficacy of bergamot‐containing nutraceuticals in weight loss at a dose ≤ 600 mg/day and in participants with overweight and obesity, results that also extend to BMI and WC (Table 2). Thus, despite the observed funnel plot asymmetry, the robustness of our findings is supported by consistent results across subgroup analyses and the high methodological quality of most included studies.

The results obtained in the present meta‐analysis are similar to those found in another meta‐analysis on the anthropometric and cardiometabolic effects of polyphenols extracted from polyphenol‐rich food sources, supplements or extracts, in participants with overweight and obesity [42]. A possible explanation for the results obtained in the present meta‐analysis is that bergamot could reduce body weight, BMI, and visceral fat thanks to its polyphenols, which exert beneficial effects on lipid metabolism and body fat regulation [42]. A recent study in mouse models [42] showed that bergamot polyphenol extracts promote the “browning” of white fat, transforming it into brown fat, which is more metabolically active. This process helps burn more calories and reduce overall body fat. Although the “browning” of white adipose tissue (WAT) induced by bergamot polyphenol extracts has been demonstrated in cellular [43] and murine models [42], direct evidence in humans remains limited. In obese mice, supplementation with a BPF significantly reduced weight gain and upregulated thermogenic markers such as UCP1, confirming the ability of these compounds to induce fat browning and improve metabolic outcomes in vivo [42]. More broadly, dietary polyphenols have been shown to enhance mitochondrial biogenesis, activate AMPK–PGC‐1α pathways, and increase energy expenditure, thereby promoting the acquisition of a brown‐like phenotype in WAT [43]. However, preliminary findings are promising. For instance, Nirengi et al. [44] showed that daily ingestion of a polyphenol‐rich beverage increased brown adipose tissue density in healthy young women, supporting the notion that polyphenols may regulate brown adipogenesis and promote WAT browning in humans. The limited number of human studies may be explained by methodological challenges in assessing BAT activity and gene expression in vivo, along with short study durations, small sample sizes, and high interindividual variability in BAT activation [45, 46]. Nevertheless, the biological plausibility of this mechanism, supported by consistent preclinical data, suggests that it may contribute, at least in part, to the reductions in body weight and WC observed in clinical trials investigating *C. bergamia* supplementation. In addition, bergamot may reduce inflammation and lipogenesis and improve fat oxidation [42, 47], thereby promoting the reduction of visceral fat, which is associated with cardiovascular and metabolic risk. Furthermore, the reduction in weight, BMI, and WC could be explained by the fact that polyphenols reduce energy intake by acting on the mechanisms regulating hunger and satiety [48]. These findings suggest that supplementation with nutraceuticals containing *C. bergamia* may be a valid aid in the management of obesity, offering a cheaper solution with fewer side effects than pharmacotherapy for obesity [49, 50]. In addition to its role in managing general obesity, recent evidence also suggests that bergamot may play a beneficial role in more complex obesity phenotypes, such as osteosarcopenic obesity [13]. In particular, the polyphenol‐rich composition of *C. bergamia* may exert positive effects on fat metabolism, muscle mass preservation, and bone health, offering promising avenues for the integrated management of metabolic disorders [13]. Furthermore, subgroup analysis showed that nutraceuticals containing *C. bergamia* led to significant reductions in body weight at dosages ≤ 600 mg/day but not at doses ≥ 1000 mg/day (Table 2). The absence of a clear dose–response relationship may be attributed to several factors. Firstly, the analysis included only three studies assessing body weight and just two for BMI at higher doses, limiting the statistical power. Secondly, the included studies involved heterogeneous populations, including both normal‐weight and overweight/obese individuals, which could dilute potential effects. Third, while direct pharmacokinetic data on bergamot polyphenols at different dosages remain limited, evidence from studies on other polyphenolic compounds suggests that oral absorption may plateau at higher doses due to saturation of intestinal uptake mechanisms [51], whereby increasing the dose beyond a certain threshold does not proportionally enhance systemic bioavailability. Lastly, the interpretation is further complicated by the variable composition of the nutraceutical formulations containing *C. bergamia* , which often include other bioactive compounds that may interact or interfere with the effects of bergamot itself.

However, it is known that high doses of polyphenols can be potentially toxic to human health, causing disorders such as nausea, vomiting, gastrointestinal dysfunction, headache, inflamed skin, tachycardia, and chest tightness [52]. Therefore, doses ≤ 600 mg/day could reduce the risk of adverse effects while maintaining efficacy in body weight control.

### 3.1 Strengths and Limitations

This meta‐analysis has several strengths, including the inclusion of clinical trials in subjects with a limited number of obesity‐related health conditions, such as hypercholesterolemia, metabolic syndrome, and nonalcoholic fatty liver disease. This reduced the influence of other conditions on the outcomes, making the results more practical and generalizable. Only randomized studies, both parallel and crossover design, were included, thus ensuring high‐quality data and inferences. Furthermore, subgroup analyses provided overlapping results that confirmed the results of the main analysis, consolidating the conclusions on changes in anthropometric parameters after supplementation of nutraceuticals containing *C. bergamia* .

However, this meta‐analysis has some limitations. The main one is the lack of homogeneity between studies, which may limit the validity of the results. Another limitation is the inclusion of normal‐weight subjects, as well as those with overweight or obesity. The duration of the studies ranged from 4 to 24 weeks, with most of them lasting 12 weeks. The short duration of treatment limits the generalizability of the beneficial effects of bergamot beyond the study period. Furthermore, it was not possible to analyze the potential effects of bergamot in the population stratified by age groups or gender. The different formulations and concentrations of polyphenols of *C. bergamia* , together with the presence of other bioactive molecules, could have enhanced or attenuated the effects of bergamot itself.

### 3.2 Future Prospects

To better understand the effect of bergamot nutraceutical supplementation in obesity management, larger and longer term studies of at least 12 months, using exclusively *C. bergamia* polyphenols, are needed. Additionally, in many of the included studies, changes in body weight and obesity‐related anthropometric parameters were not the primary objectives. Therefore, to obtain more robust conclusions on the anti‐obesity benefits of *C. bergamia* nutraceuticals, RCTs specifically designed to evaluate the effects of bergamot polyphenol supplementation in adults with overweight or obesity are required.

## 4 Conclusions

Obesity is a global pandemic with serious health impacts and high economic costs. This systematic review and meta‐analysis demonstrate that bergamot supplementation may be effective in preventing and managing obesity in adults with metabolic complications. The findings indicate that the intake of nutraceuticals containing *C. bergamia* can significantly reduce body weight, BMI, and WC. In conclusion, nutraceuticals containing bergamot could be used as valid aids in the management of obesity when combined with lifestyle modifications. However, further ad hoc studies are needed to confirm these results.

## Author Contributions

All authors have read and approved the final manuscript.

## Funding

This study was supported by grants from Italy's Development and Cohesion Fund 2014–2020, provided by the Italian Ministry of Health (Grant Number: NutriDieMMe Project, T5‐AN‐14).

## Conflicts of Interest

The authors declare no conflicts of interest.

## Supporting information


**Figure S1:** Funnel plot for changes in mean body weight, BMI, waist circumference, and fat mass percentage.

## Data Availability

The data that support the findings of this study are available from the corresponding author upon reasonable request.
