# Supplementary material for: Effect of Citrus bergamia Supplementation on Body Composition in Humans: A Systematic Review and Meta‐Analysis of Randomized Controlled Trials
Source: Obes Rev. 2026 Jan 22;27(7):e70094. doi: 10.1111/obr.70094 (PMC13243342; doi:10.1111/obr.70094)
Supplement: Supplementary file 1 — Figure S1: Funnel plot for changes in mean body weight, BMI, waist circumference, and fat mass percentage. [file OBR-27-e70094-s001.pdf]

Title: Effect of *Citrus bergamia* supplementation on body composition in humans: A Systematic review and Meta-analysis of randomized controlled trials

Running title: Impact of *Citrus bergamia* on Body Composition: Systematic Review and Meta-analysis

Carmelo Pujia[1*], Yvelise Ferro[2*], Alberto Castagna[2], Elisa Mazza[3], Samantha Maurotti[3], Francesca Rita Noto[2], Valeria Rizzo[3], Tiziana Montalcini[3,4#], Arturo Pujia[2,4]

[1] O.U. Clinical Nutrition, Renato Dulbecco Hospital, 88100 Catanzaro, Italy

[2] Department of Medical and Surgical Sciences, University "Magna Græcia" of Catanzaro, 88100 Catanzaro, Italy

[3] Department of Clinical and Experimental Medicine, University "Magna Græcia" of Catanzaro, 88100 Catanzaro, Italy

[4] Research Center for the Prevention and Treatment of Metabolic Diseases, University "Magna Græcia", 88100 Catanzaro, Italy

* Equal contribution

# Corresponding Author:

Prof.ssa Tiziana Montalcini

email: tmontalcini@unicz.it

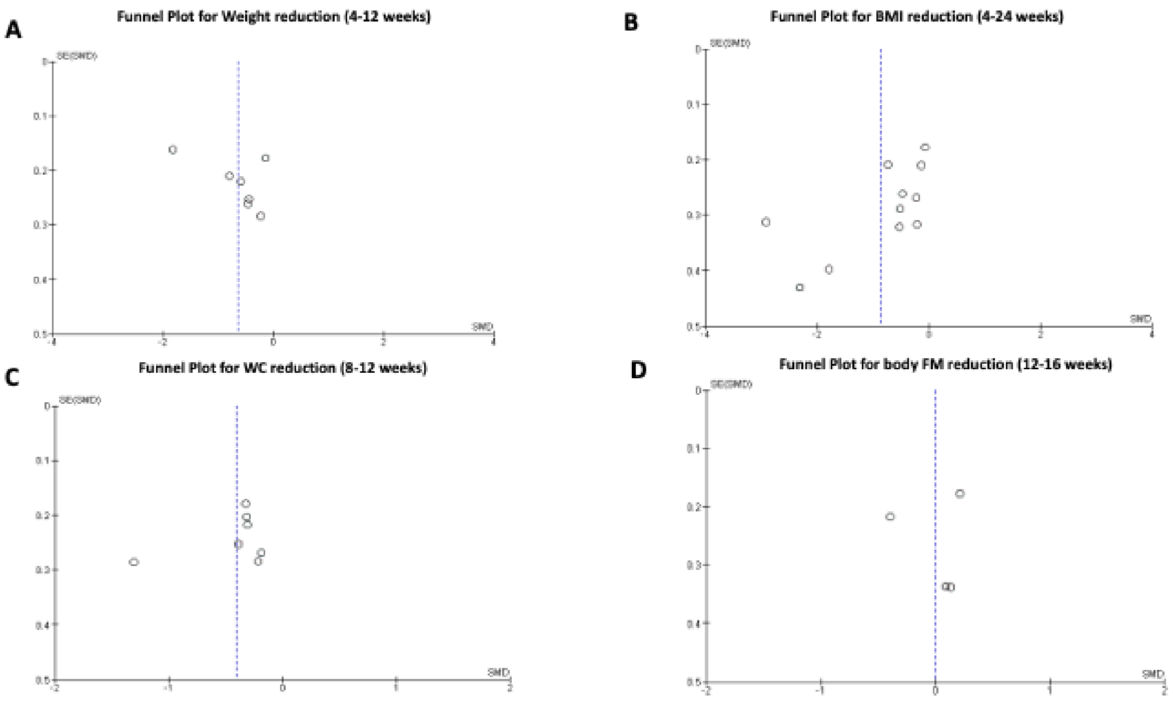

Supplemental figure 1. Funnel plot for changes in mean body weight, BMI, waist circumference and fat mass percentage
